# Transarterial chemoembolization for unresectable hepatocellular carcinoma: A comparison of the efficacy and safety of 2 embolic agents

**DOI:** 10.1097/MD.0000000000010832

**Published:** 2018-05-25

**Authors:** Shiguang Chen, Wenchang Yu, Kongzhi Zhang, Weifu Liu, Qizhong Chen

**Affiliations:** Department of Interventional Oncology, Fujian Cancer Hospital & Fujian Medical University Cancer Hospital, Fuzhou, China.

**Keywords:** chemoembolization, embolic agents, ethiodized oil, gelatin sponge particle, hepatocellular carcinoma

## Abstract

The aim of this study was to compare the efficacy and safety of 2 different embolic agents, namely gelatin sponge particle (GSP) and Lipiodol, for transarterial chemoembolization (TACE) of unresectable hepatocellular carcinoma (HCC).

We retrospectively reviewed 87 consecutive patients with unresectable HCC who underwent Lipiodol TACE with lobaplatin and 87 consecutive patients with unresectable HCC who underwent GSP TACE with lobaplatin between January 2013 and June 2017 in our institution as the initial treatment. Both groups were compared considering the clinical and laboratory outcomes and imaging findings before and after TACE. Tumor response and adverse events were also evaluated.

There was significant difference in the rate of complete and overall response between the groups (*P* = .029 and .001, respectively), specifically when the tumor size was >5 cm (*P* = .001). The disease control rate was significantly better in the GSP group than in the Lipiodol group (94.3% vs. 86.4%, *P* = .011). The response differences in higher stages were significant between the 2 groups (*P* = .035 and .007, respectively). The grades of adverse events were also significantly different between the groups (*P* = .000).

GSP—as an embolic agent in TACE for HCC—could significantly increase the rate of tumor response 1 month after treatment, especially in large tumors, without any significant increase in severe adverse events, when compared to Lipiodol.

## 1 Introduction

As one of the top 10 cancers worldwide, hepatocellular carcinoma (HCC) has a poor prognosis, with only a minority of patients being candidates for curative measures such as liver transplantation, hepatic resection, and percutaneous ablation.^[1,2]^ In the majority of cases, HCC is first diagnosed at an advanced stage, and such patients will only be eligible for palliative therapy. Transarterial chemoembolization (TACE) remains the treatment of choice in the management of patients with surgically unresectable HCC,^[3,4]^ although some meta-analyses reported no difference in survival between embolization alone and chemoembolization.^[5,6]^ TACE is widely used worldwide in treating advanced HCC, and there is a need for a universal standard treatment protocol,^[7]^ as different chemotherapeutic regimens, different embolic agents, and technical details may influence patient outcome.^[8]^ Gelatin sponge particles (GSP) are commonly used as adjunctive embolization agents for HCC in conventional transarterial chemoembolization (c-TACE).^[9,10]^ Kamran et al^[11]^ reported that the use of GSP as an embolization agent alone in TACE could obtain a safe and effective result. The most popular TACE technique is a chemotherapeutic agent-in-lipiodol emulsion, followed by embolic agents. Lipiodol can be used not only as a carrier of chemotherapeutic drugs in tumor, but also as a embolization agent for tumor vessels.^[12,13]^ In this study, we aimed to compare the outcome of different embolic agents, namely GSP and Lipiodol(iodized oil), considering their efficacy and safety in the treatment of unresectable HCC.

## 2 Materials and methods

### 2.1 Ethics statement

The study was approved by the Ethics Committee of Fujian Cancer Hospital, China. All patients provided written informed consent.

### 2.2 Patient selection

We usually recommended Lipiodol or GSP as an embolization agent for TACE in patients with unresectable HCC in our institution. The most common reason for the rejecting the use Lipiodol is the high cost, as Lipiodol (Guerbet Laboratories, Aulnay-Sous-Bois, France) is not on the list of agents covered by medical insurance in China and patients cannot afford it. For those who refused Lipiodol, GSP was recommended as an embolization agent in TACE. We retrospectively reviewed 87 consecutive patients with unresectable HCC who underwent Lipiodol TACE with lobaplatin and 87 consecutive patients with unresectable HCC who underwent GSP TACE with lobaplatin between January 2013 and June 2017 in our institution as initial treatment. Patients were diagnosed with surgically unresectable HCC by a multidisciplinary HCC professional group of surgeons, interventional oncologists, radiologists, and oncologists. In half of these patients, Lipiodol was administered as the embolic agent and in the other half, the embolic agent administered was GSP. The sex distribution was 80 men and 7 women in the Lipiodol group, and 72 men and 15 women in the GSP group, with a mean age of 52.7 ± 9.2years and 55.4 ± 27.6years, respectively.

The diagnosis of HCC was established based on the recommendations of the European Association for the Study of the Liver^[14]^ and the American Association for the Study of Liver Disease^[15]^ using clinical data, contrast-enhanced magnetic resonance imaging (MRI) or computed tomography (CT) findings, and increased serum level of alpha-fetoprotein (AFP). In 37 patients (21%), the diagnosis was also confirmed using ultrasound or CT-guided fine-needle biopsy findings. Patients with unresectable HCC were included in our study, irrespective of evidence of vascular invasion (including portal, hepatic vein, and inferior vena cava thrombosis) or extrahepatic metastasis. Other inclusion criteria were as follows: Eastern Cooperative Oncology Group performance status of 0 to 2; Child-Pugh class A or B; appropriate hematologic and liver function (leukocytes ≥3000 cells/mm^3^, platelets ≥50,000 cells/mm^3^, serum total bilirubin ≤2 mg/dL).

A week before treatment, we collected a comprehensive medical history and measured serum alanine aminotransferase (ALT), aspartate aminotransferase (AST), total bilirubin (TBil), albumin (ALB), prothrombin time (PT), and AFP levels, as well as determined the hepatitis B surface antigen (HBsAg) status. Abdominal contrast-enhanced MRI or CT and chest CT were also part of the initial workup in all patients.

### 2.3 Materials

For embolization, we used Lipiodol (Guerbet Laboratories, Aulnay-Sous-Bois, France) or GSP (Yiling Pharmaceutical Co., Ltd. Hangzhou, China). The GSP was available in 3 size ranges (150–350 μm, 350–560 μm, and 560–710 μm). We used lobaplatin (Hainan Changan International Pharmaceutical Co., Ltd. Hainan, China) as the chemotherapy agent.

### 2.4 TACE procedures

In all patients who underwent TACE, the Seldinger technique was followed for hepatic artery catheterization. Using digital subtraction angiography, the catheter or microcatheter was inserted from the right femoral artery and guided to the hepatic artery or its branches for angiography. Tumor-feeding arteries were then superselected based on our understanding of the tumor blood supply, revealed via hepatic arteriography. An emulsion of the embolic agent and 50 mg of lobaplatin was then injected in the tumor-feeding arteries. The dose of the embolic agent was determined by the size and number of the tumor, and ranged from 3 to 30 mL for Lipiodol (14.61 ± 1.41 mL) and 25 to 130 mg for GSP (78.4 ± 38.9 mg). In TACE with GSP, the embolic emulsion also contained 30 ml of contrast medium. The size of GSP depended on the superselected hepatic artery and tumor size, and the most common size used was in the range of 350 to 560 μm. In 8 patients (9.2%) with a small size tumor (≤5 cm) and a clear boundary, the microcatheter was inserted in the tumor gate; hence, we used smaller-size GSP for treatment. The lesions were too large to be embolized completely in 7 patients (8.0%), and we chose GSP size of 560 to 710 μm. The aim of chemoembolization was to achieve complete arterial blockage in the arteries supplying the tumor. In some patients with serious portal thrombosis, wide tumor distribution, hepatic arterioportal fistula (APF), or hepatic arterial venous fistula (AVF), the full doses of embolic agents were not administered because of the high risk for failure of recovery of liver function after treatment. There were 16 patients (18.2%) in the GSP group and 9 patients (10.3%) in the Lipiodol group who were not candidates for complete embolization, although this difference was not significant (*P* = .194). There was also no significant difference in the distribution of patients with APF or AVF between the 2 groups (7 [8%] vs. 9 [9.2%]; *P* = 1.000]).

### 2.5 Assessment of tumor response

Contrast-enhanced MRI or CT was performed 1 month after transcatheter arterial chemoembolization, and the results were evaluated according to the modified Response Evaluation Criteria in Solid Tumors (mRECIST) to assess tumor response.^[16]^ Based on the results, patients were divided into the following 4 categories: complete response (CR),which was described as the disappearance of intratumoral arterial enhancement in all target lesions; partial response (PR), defined as a reduction of at least 30% in the sum of the diameter of the live (arterial phase) target lesions compared with the baseline diameter of the target lesions; stable disease (SD), including any case that did not meet the criteria for PR or progressive disease (PD); PD, defined as an increase of at least 20% in the sum of the diameter of residual (enhanced) target lesions compared with the minimum sum of the diameter of live (enhanced) target lesions at the beginning of treatment. The rate of overall response (OR) was calculated as the rate of CR plus PR. Disease control rate (DCR) was calculated as the sum of the best response ratings in CR, PR, and SD.

### 2.6 Toxicity assessments

One week after TACE, hematologic and liver function tests were performed in all patients and any dysfunction was evaluated. Treatment-related adverse events (AEs) in the patients enrolled in this study included fever, abdominal pain, nausea, vomiting, diarrhea, constipation, cholecystitis, and infection within 1 month after interventional treatment. The grading of any AE was in accordance with the National Cancer Institute Common Terminology Criteria for adverse events, version 3.0.^[17]^

### 2.7 Statistical analysis

All statistical analyses were carried out using the SPSS software (version 18.0, SPSS, Chicago, IL). To evaluate the significance of the differences between the 2 groups, the *χ*^2^ test and independent samples *t* test were used. *P* < .05 indicated a significant difference.

## 3 Results

### 3.1 Patient characteristics

After statistical analysis, we did not observe any significant difference in the distribution of age; sex; expression of HBsAg; ALT, AST, TBil, ALB, PT, and AFP levels; Child-Pugh class; maximum HCC size; number of HCC foci; Barcelona Clinic Liver Cancer (BCLC) stage; extrahepatic metastasis; vascular invasion; and APF/AVF between the 2 groups(Table 1).

**Table 1:**
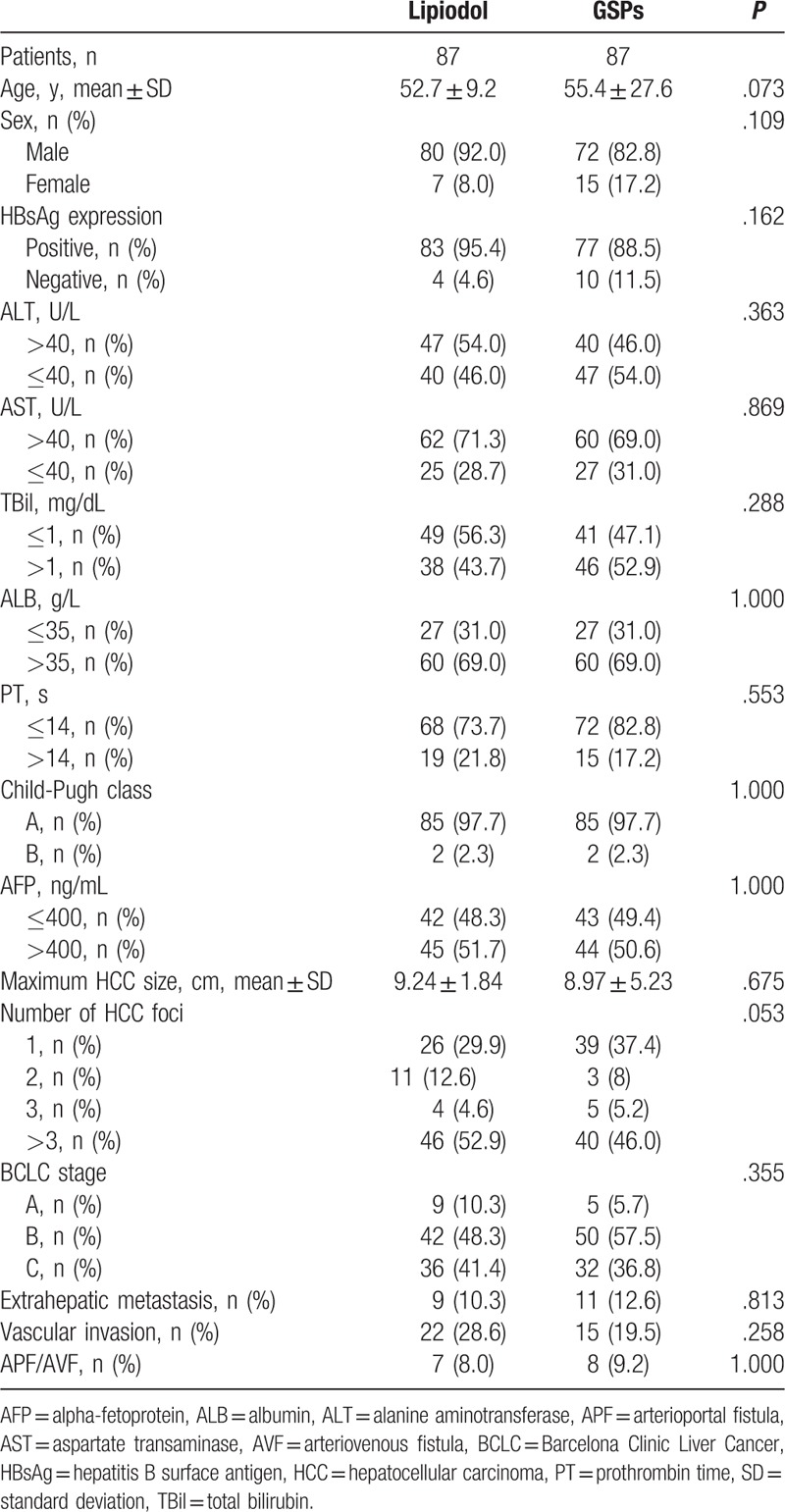
Baseline characteristics of patients undergoing the TACE procedure.

### 3.2 Tumor response

**Figure 1:**
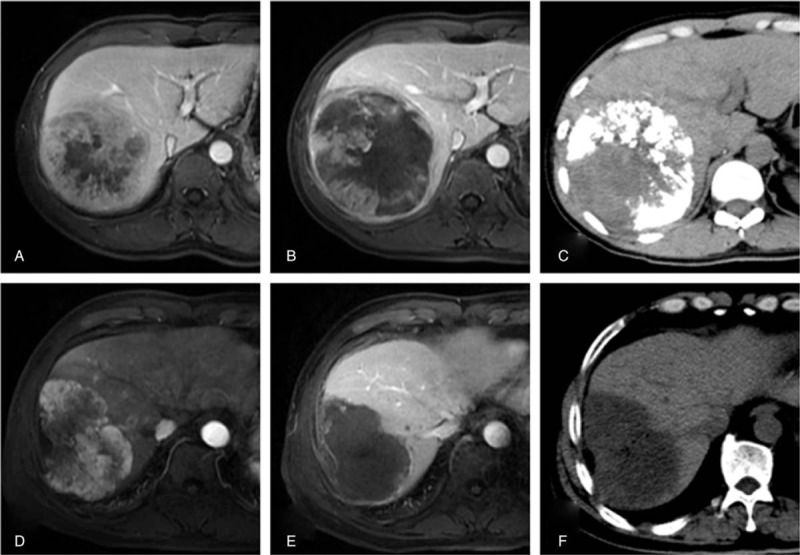
Results of abdominal computed tomography (CT) and magnetic resonance imaging (MRI) and the changes in tumors in a representative case before and after transarterial chemoembolization (TACE). MRI with tumor enhancement pre-TACE in a patient later treated with GSP (A) and Lipiodol (D). Note the enhanced integrity of the tumor in both A and D. MRI with tumor enhancement and CT scan in the patient treated with Lipiodol (B and C) and gelatin sponge particle (E and F). Partial enhancement can be seen in Figure B. There is no enhancement in the tumor in Figure E, the distribution of Lipiodol in the tumor is shown in Figure C. A low-density shadow is found in the tumor (F).

Based on the results of abdominal MRI or CT scans performed 1 month after TACE, the changes in tumors were different between the 2 groups (Fig. 1A–F).

We also evaluated tumor response in all 154 patients in both groups using mRECIST, with the results presented in Table 2. CR was observed in 5 patients (5.7%) in the GSP group, but not in the Lipiodol group, which was significantly different between the 2 groups (*P* = .029). The OR (CR + PR) was significantly higher in the group treated with GSP than in the group treated with Lipiodol (59 [67.8%] vs. 37 [42.5%]; *P* = .001). The DCR (CR + PR + SD) was also better in the GSP treated patients than in patients who had received Lipiodol (94.3% vs. 86.4%; *P* = .011).

**Table 2:**
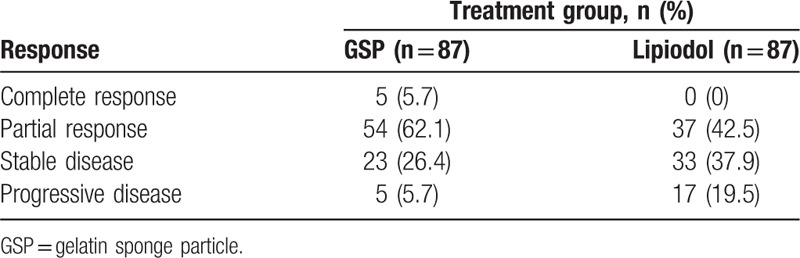
Response evaluation in the GSP and Lipiodol groups.

Further analysis showed that the OR between the 2 groups in the BCLC stage A was not significantly different, but this was not the case in patients with BCLC stages B and C disease (*P* *=* .035 and .007, respectively). When the size of the tumor exceeded 5 cm, patients treated with GSP had a significantly better OR than those treated with Lipiodol (*P* = .001). Such a difference was not observed in patients with smaller tumors(*P* = .321). The results are presented in Table 3.

**Table 3:**
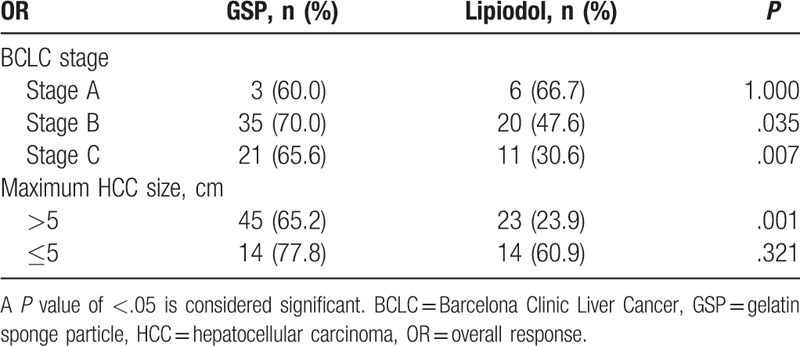
OR in the GSP and Lipiodol groups.

### 3.3 Adverse events

There were no treatment-related deaths within 1 month of the current study. The AEs associated with the interventional treatment observed in the 2 groups are listed in Table 4. No grade 4 AE was reported. The most common AEs were hematologic toxicity, post-embolization syndrome (including fever, abdominal pain, nausea, and vomiting), and liver dysfunction. Grade 3 ALT levels and abdominal pain occurred in 2 (2.3%) and 5 (6.5%) patients in the GSP group, respectively. Grade 3 anemia was observed in 2 (2.3%) patients in the Lipiodol group. In the GSP group, ALT levels of grades 1, 2, and 3 were noted in 28 (32.2%), 20 (23.0%), and 2 (2.3%) patients, respectively, whereas 15 (17.2%) and 4 (4.6%) patients in the Lipiodol group had ALT levels of grades 1 and 2. This difference was significant between the 2 groups (*P* = .000). The difference in the occurrence of abdominal pain was also significant between the 2 groups (*P* = .000), with grade 1 abdominal pain observed in 40 (51.9%), grade 2 in 15 (15.9%), and grade 3 in 5 (6.5%) patients in the GSP group, whereas grade 1 abdominal pain was reported in 4 (5.2%) patients treated with Lipiodol and grade 2 in 8 (10.4%) patients. One case of infection (such as septicemia) was noted in the Lipiodol group that resolved after 1 week of antibiotic treatment.

**Table 4:**
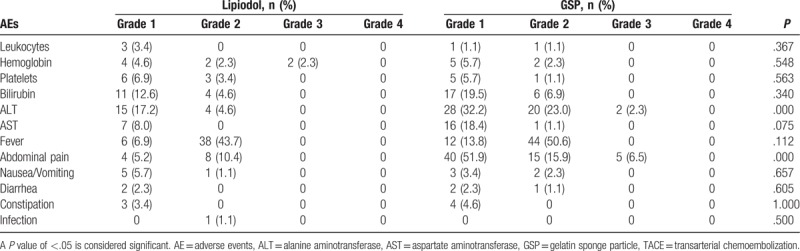
Treatment-related AEs in patients undergoing TACE.

## 4 Discussion

Currently, there is no criterion standard for the selection of arterial embolic agents in clinical practice. In addition, the use of different embolic agents has not been reported to have any significant effect on survival.^[8,18]^Arterial embolic agents are mainly divided into permanent embolic agents and absorbable embolic agents. As a conventional permanent embolic agent, Lipiodol is commonly used in arterial embolization, and has been proved to be effective in unresectable HCC.^[4]^ GSP is anabsorbable embolic agent, with the recanalization of the embolized hepatic arteries observed 3 weeks after treatment in 70% to 80% of patients,^[19,20]^leading to less liver dysfunction after treatment.^[21]^

Overall survival is the primary endpoint in cancer research, and we initially assessed tumor response according to the WHO criteria,^[22]^ wherein both CR and PR are considered as effective response.^[23]^ In our study, GSP was compared with iodide oil, as embolic agents, 1 month after TACE, and we found significant differences in CR, OR, and DCR between the 2 treatment groups. This might be because GSP embolization of tumor-feeding arteries is more complete, leading to tumor necrosis in a shorter time and a higher CR, making the embolization of the target lesion more efficient.

TACE can improve survival in strictly selected patients with unresectable HCC.^[3]^ Several studies have suggested that asymptomatic multinodular neoplasms without vascular invasion or extrahepatic tumor diffusion, wherein liver function has been conserved, were the best choice for TACE treatment in patients with HCC.^[1,24,25]^ There was no significant difference in the use of either embolic agent in patients with BCLC stage A, but the GSP group had a significantly better OR in stages B and C (*P* = .035, .007, respectively). The OR was also significantly higher in the GSP group, when the maximum size of the tumor exceeded 5 cm (*P* = .001). Chen et al^[12]^ reported that high-dose Lipiodol chemoembolization, used in the treatment of large HCC (the largest diameter of the lesion exceeded 5 cm), was practically acceptable and had a good outcome. In our study, we found that the use of GSP in arterial embolism is better than iodide oil in patients with large HCC. The reason might be that either the use of GSP embolization in large HCC lesions can block the tumor-feeding arteries more thoroughly, or the embolic agent is not easily removed from the target lesions after embolization, resulting in a longer delay in the recanalization of the tumor's blood vessels. To the best of our knowledge, there have been no reports comparing the outcome of chemoembolization with GSP or Lipiodol to date. The findings of this study may provide some clarification regarding whether GSP or Lipiodol should be used for patients with HCC, especially for patients with large tumors.

No serious complications were found in any patient in the present study. However, in the GSP group, abdominal pain and liver dysfunction (according to serum ALT levels) were more frequently reported than that in the Lipiodol group (*P* = .000). Most adverse events observed in the patients in our study were of grades 1 and 2, and patients recovered after simple treatment. More severe tumor necrosis (hence, a higher OR in patients) was noted after GSP embolization than Lipiodol. GSP is used as a temporary embolic agent in TACE, as the hepatic arteries will recanalize shortly after embolization. In general, GSP, as an arterial embolic agent in TACE treatment, is as safe as Lipiodol.

Doxorubicin, mitomycin, fluorouracil, and cisplatin are commonly used as chemotherapeutic agents in the treatment of TACE worldwide. Nonselective TACE with cisplatin and GSP were found to be effective in multifocal HCC at the recommended dose.^[26]^ TACE using a suspension of cisplatin in lipiodol may be a useful treatment in patients with HCC.^[27]^ However, the dose of cisplatin was limited by renal, gastrointestinal, and neurological toxicities.^[28]^ In the present study, we used lobaplatin as a chemotherapeutic agent to replace cisplatin, which mixed with lipiodol or GSP. Lobaplatin arrests cell cycle progression in G1 and G2/M phase and may inhibit proliferation of human HCC cells.^[29]^ Furthermore, loplatin, a novel platinum analogue, was less toxic and less resistant than cisplatin.^[30]^

Three limitations should be considered in our study. First, it was not randomized, so the choice of selective or nonselective procedures was determined by the tumor or the condition of the liver, despite the similarity in patient characteristics between the 2 patient groups. Second, our study is probably biased in the selection of patients considering its retrospective nature and that it was carried out in a single center. Third, the tumor response was only the initial observation endpoint, and we did not perform follow-up tests to determine the risk for progression and overall survival rate.

## Author contributions

**Conceptualization:** Shiguang Chen, Wenchang Yu.

**Formal analysis:** Shiguang Chen.

**Investigation:** Kongzhi Zhang, Weifu Liu, Qizhong Chen.

**Methodology:** Shiguang Chen.

**Supervision:** Wenchang Yu.

**Validation:** Shiguang Chen.

**Writing – original draft:** Shiguang Chen, Kongzhi Zhang, Weifu Liu, Qizhong Chen.

**Writing – review & editing:** Shiguang Chen, Wenchang Yu.

## Acknowledgments

The authors thank all the patients and their families for allowing us to carry out this study. The project was supported by the National Clinical Key Specialty Construction Program of China.
